# Supplementary material for: Seminoma and Embryonal Carcinoma Footprints Identified by Analysis of Integrated Genome-Wide Epigenetic and Expression Profiles of Germ Cell Cancer Cell Lines
Source: PLoS One. 2014 Jun 2;9(6):e98330. doi: 10.1371/journal.pone.0098330 (PMC4041891; doi:10.1371/journal.pone.0098330)
Supplement: File S1 — ZIP file containing DMRforPairs output for significant regions. Please start from the html files. (ZIP) [file pone.0098330.s008.zip › figures/33643.pdf]

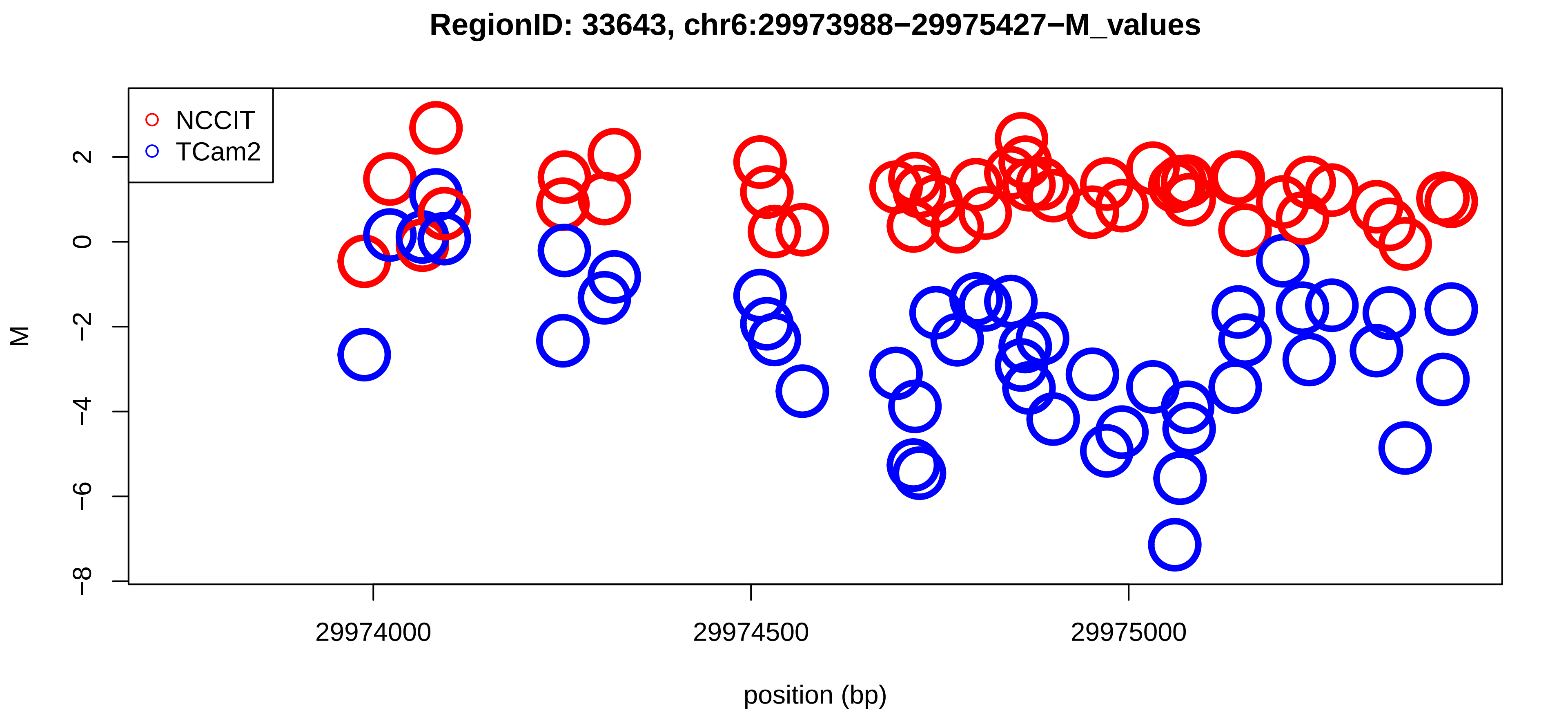

RegionID: 33643, chr6:29973988−29975427−M_values
NCCIT
TCam2
M
2
0
−2
−4
−6
−8
29974000
29974500
29975000
position (bp)

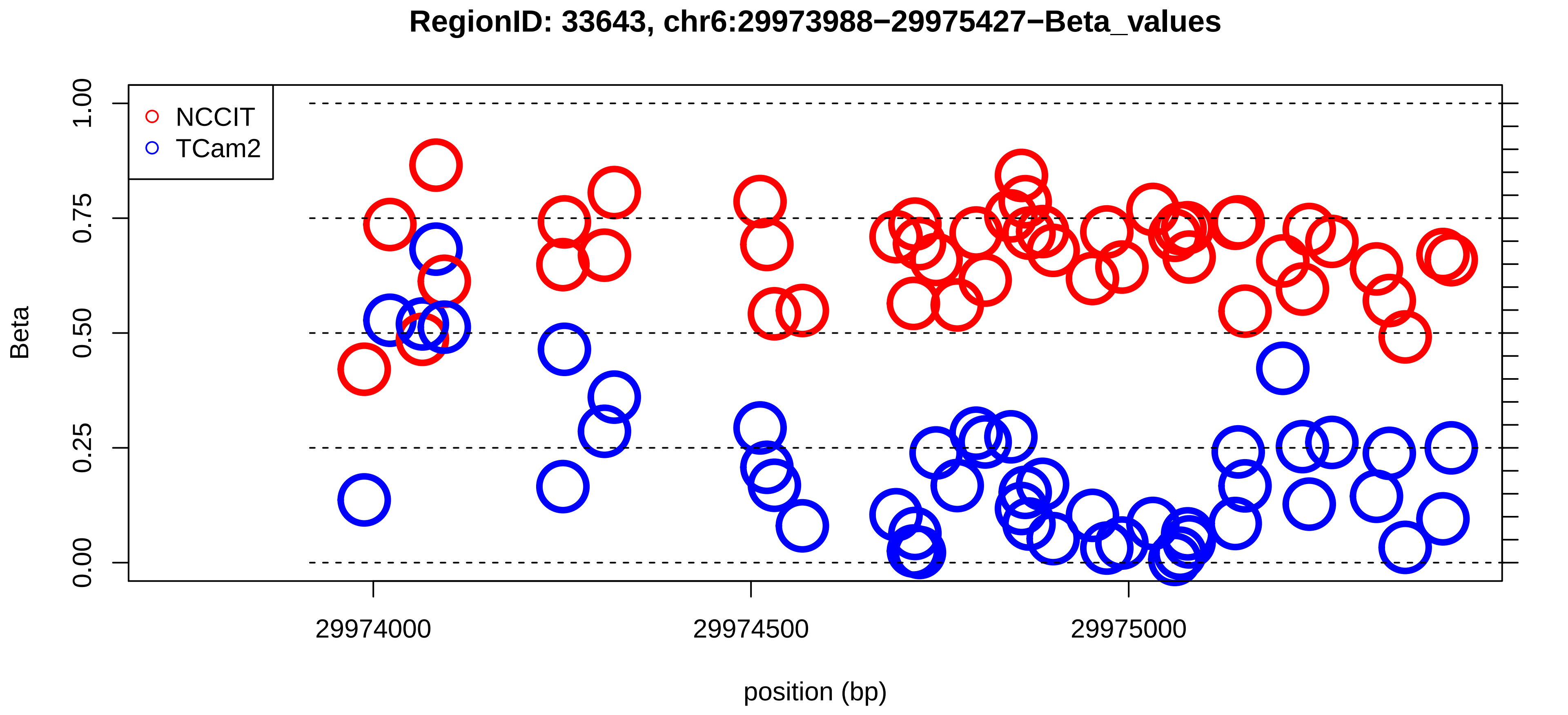

RegionID: 33643, chr6:29973988−29975427−Beta_values
NCCIT
TCam2
Beta
1.00
0.75
0.50
0.25
0.00
29974000
29974500
29975000
position (bp)

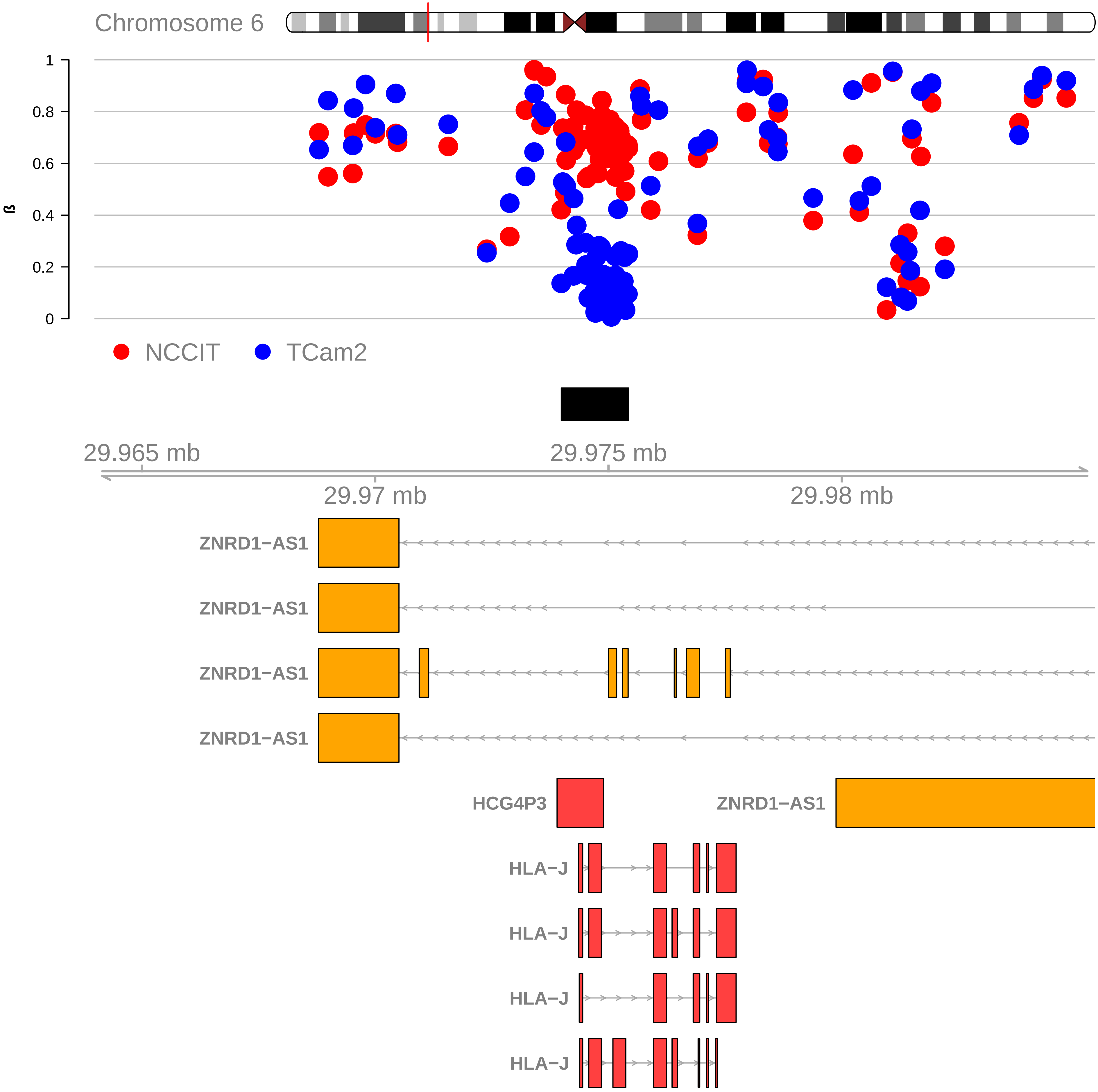

Chromosome 6
ß
1
0.8
0.6
0.4
0.2
0
NCCIT
TCam2
29.965 mb
29.975 mb
29.97 mb
29.98 mb
ZNRD1−AS1
ZNRD1−AS1
ZNRD1−AS1
ZNRD1−AS1
HCG4P3
ZNRD1−AS1
HLA−J
HLA−J
HLA−J
HLA−J
